# Supplementary figures and images for: Structural and regulatory insights into the glideosome-associated connector from Toxoplasma gondii
Source: eLife. 2023 Apr 4;12:e86049. doi: 10.7554/eLife.86049 (PMC10125020; doi:10.7554/eLife.86049)

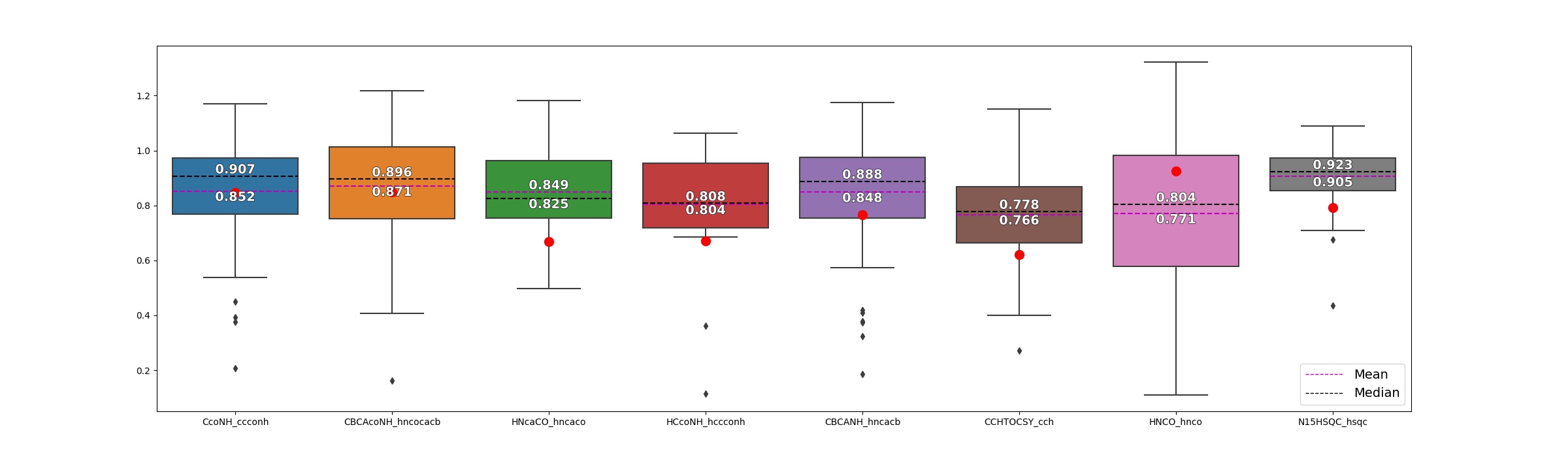

Supplement: Figure 1—source data 1. — wwPDB X-ray structure validation; MTZ file for TgGAC X-ray diffraction data and final refined PDB file for TgGAC from X-ray diffraction. [file elife-86049-fig1-data1.zip › Figure 1-source data 1/NOE assignment and NMR structure/peak_picking_plot.jpg]

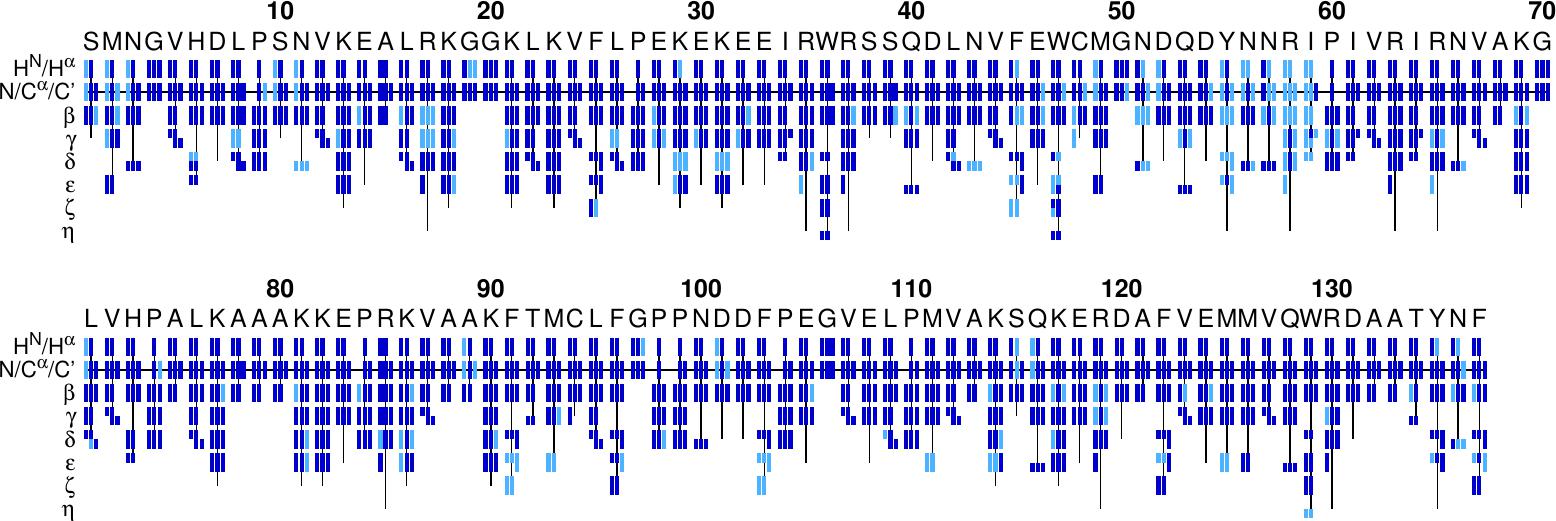

Supplement: Figure 1—source data 1. — wwPDB X-ray structure validation; MTZ file for TgGAC X-ray diffraction data and final refined PDB file for TgGAC from X-ray diffraction. [file elife-86049-fig1-data1.zip › Figure 1-source data 1/NOE assignment and NMR structure/shift_assignment/flya_plot.jpg]

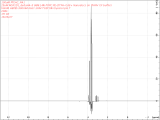

Supplement: Figure 4—source data 2. [file elife-86049-fig4-data2.zip › Figure 4-source data 2/PfGAC_N41_MSP1D1_deltaH4-5Nanodiscs_HSQC_titration/9/pdata/1/thumb.png]

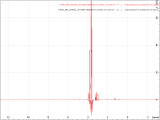

Supplement: Figure 4—source data 2. [file elife-86049-fig4-data2.zip › Figure 4-source data 2/PfGAC_N41_MSP1D1_deltaH4-5Nanodiscs_HSQC_titration/11/pdata/1/thumb.png]

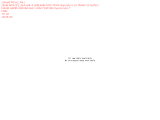

Supplement: Figure 4—source data 2. [file elife-86049-fig4-data2.zip › Figure 4-source data 2/PfGAC_N41_MSP1D1_deltaH4-5Nanodiscs_HSQC_titration/7/pdata/1/thumb.png]

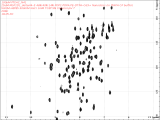

Supplement: Figure 4—source data 2. [file elife-86049-fig4-data2.zip › Figure 4-source data 2/PfGAC_N41_MSP1D1_deltaH4-5Nanodiscs_HSQC_titration/6/pdata/1/thumb.png]

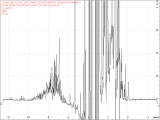

Supplement: Figure 4—figure supplement 1—source data 2. [file elife-86049-fig4-figsupp1-data2.zip › Figure S4-source data 2/TgGAC_N37_MSP1D1_deltaH4-5Nanodisc_HSQC_titration/7/pdata/1/thumb.png]

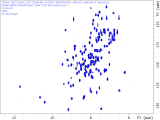

Supplement: Figure 4—figure supplement 1—source data 2. [file elife-86049-fig4-figsupp1-data2.zip › Figure S4-source data 2/TgGAC_N37_MSP1D1_deltaH4-5Nanodisc_HSQC_titration/6/pdata/1/thumb.png]

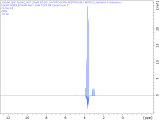

Supplement: Figure 4—figure supplement 1—source data 2. [file elife-86049-fig4-figsupp1-data2.zip › Figure S4-source data 2/TgGAC_N37_MSP1D1_deltaH4-5Nanodisc_HSQC_titration/1/pdata/1/thumb.png]

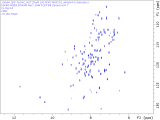

Supplement: Figure 4—figure supplement 1—source data 2. [file elife-86049-fig4-figsupp1-data2.zip › Figure S4-source data 2/TgGAC_N37_MSP1D1_deltaH4-5Nanodisc_HSQC_titration/8/pdata/1/thumb.png]

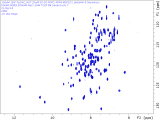

Supplement: Figure 4—figure supplement 1—source data 2. [file elife-86049-fig4-figsupp1-data2.zip › Figure S4-source data 2/TgGAC_N37_MSP1D1_deltaH4-5Nanodisc_HSQC_titration/4/pdata/1/thumb.png]

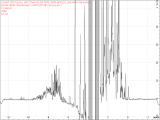

Supplement: Figure 4—figure supplement 1—source data 2. [file elife-86049-fig4-figsupp1-data2.zip › Figure S4-source data 2/TgGAC_N37_MSP1D1_deltaH4-5Nanodisc_HSQC_titration/3/pdata/1/thumb.png]

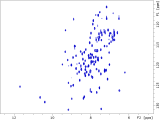

Supplement: Figure 4—figure supplement 1—source data 2. [file elife-86049-fig4-figsupp1-data2.zip › Figure S4-source data 2/TgGAC_N37_MSP1D1_deltaH4-5Nanodisc_HSQC_titration/2/pdata/1/thumb.png]

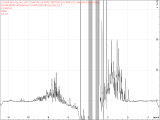

Supplement: Figure 4—figure supplement 1—source data 2. [file elife-86049-fig4-figsupp1-data2.zip › Figure S4-source data 2/TgGAC_N37_MSP1D1_deltaH4-5Nanodisc_HSQC_titration/5/pdata/1/thumb.png]

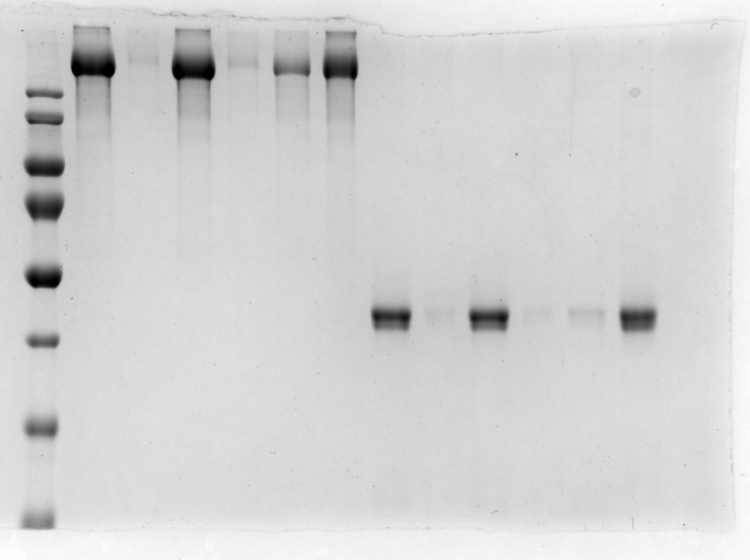

Supplement: Figure 5—source data 1. [file elife-86049-fig5-data1.zip › Figure 5-source data 1/A-GACFL-GACPH.png]

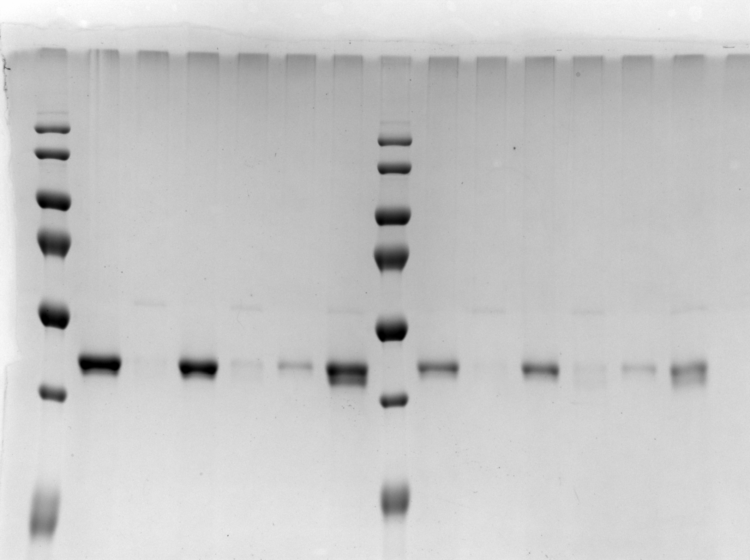

Supplement: Figure 5—source data 1. [file elife-86049-fig5-data1.zip › Figure 5-source data 1/C-GACPH-WT - GACPH-RKmut.png]

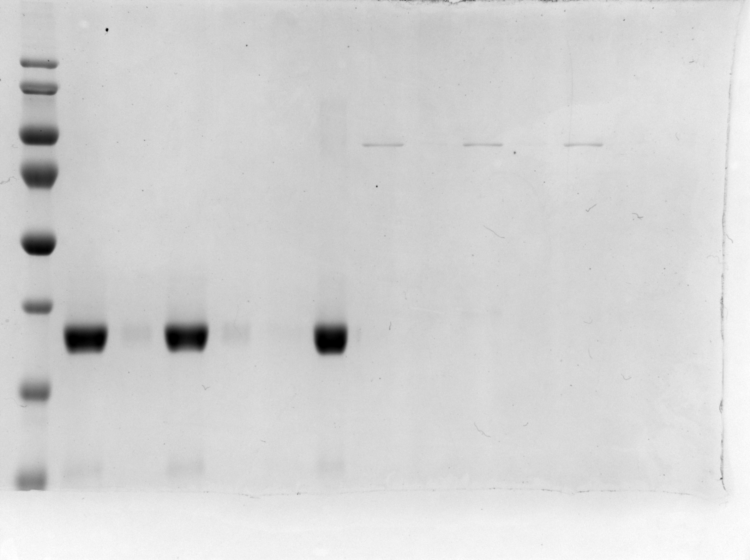

Supplement: Figure 5—source data 1. [file elife-86049-fig5-data1.zip › Figure 5-source data 1/A-APHPH.png]

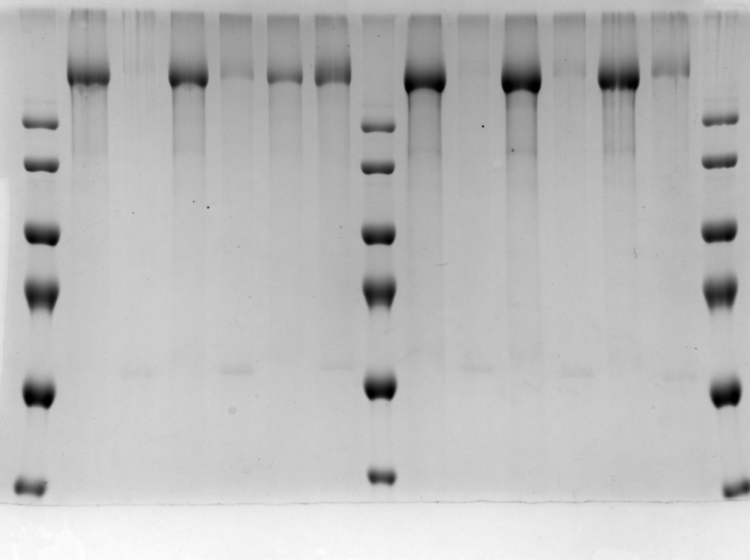

Supplement: Figure 5—source data 1. [file elife-86049-fig5-data1.zip › Figure 5-source data 1/B-GACFL-KERmut - GACFL-RKKERmut.png]

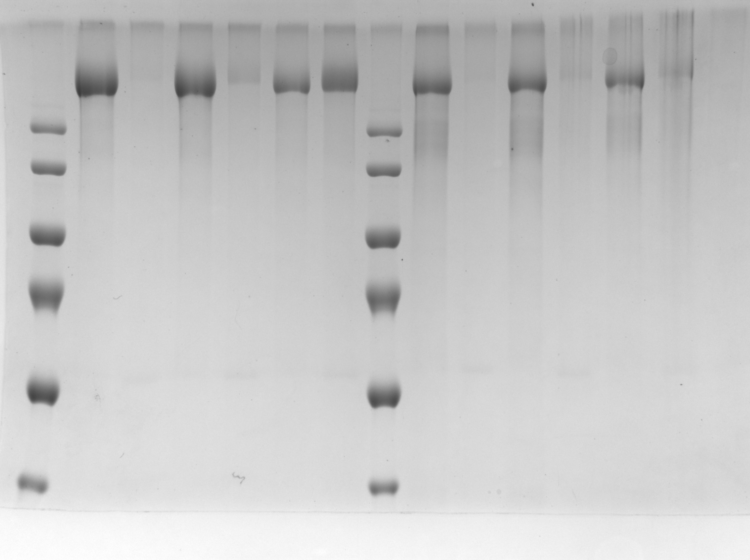

Supplement: Figure 5—source data 1. [file elife-86049-fig5-data1.zip › Figure 5-source data 1/B-GACFL-WT - GACFL-RKmut.png]

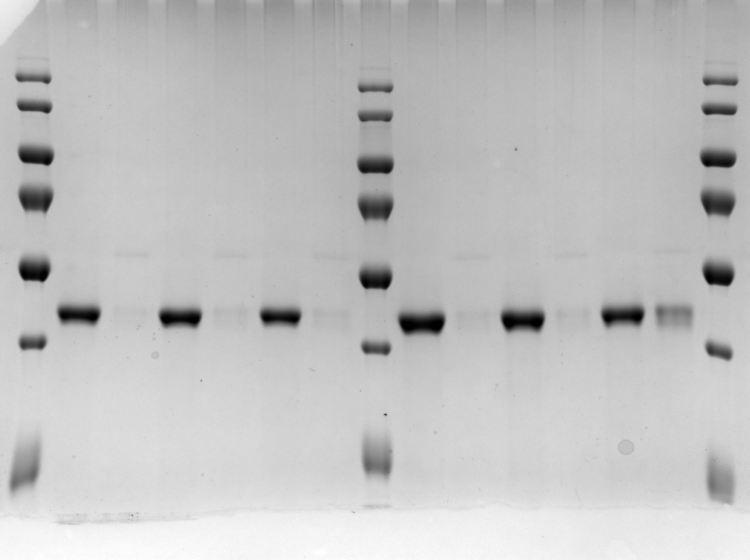

Supplement: Figure 5—source data 1. [file elife-86049-fig5-data1.zip › Figure 5-source data 1/C-GACPH-KERmut - GACPH-RKKERmut.png]

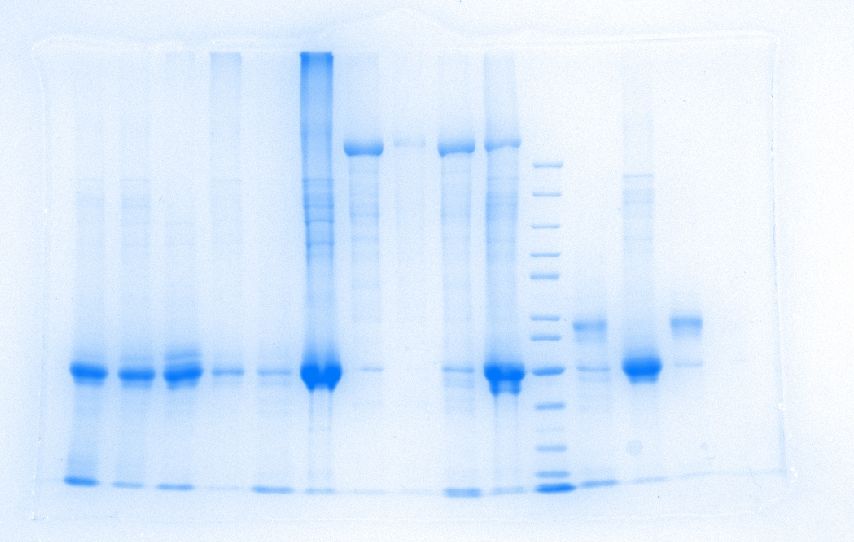

Supplement: Figure 7—source data 1. [file elife-86049-fig7-data1.zip › Figure 7-source data 1/Coomassie_Raw_TgFactin_FL_GAC_CoSedimentation_Left.jpg]

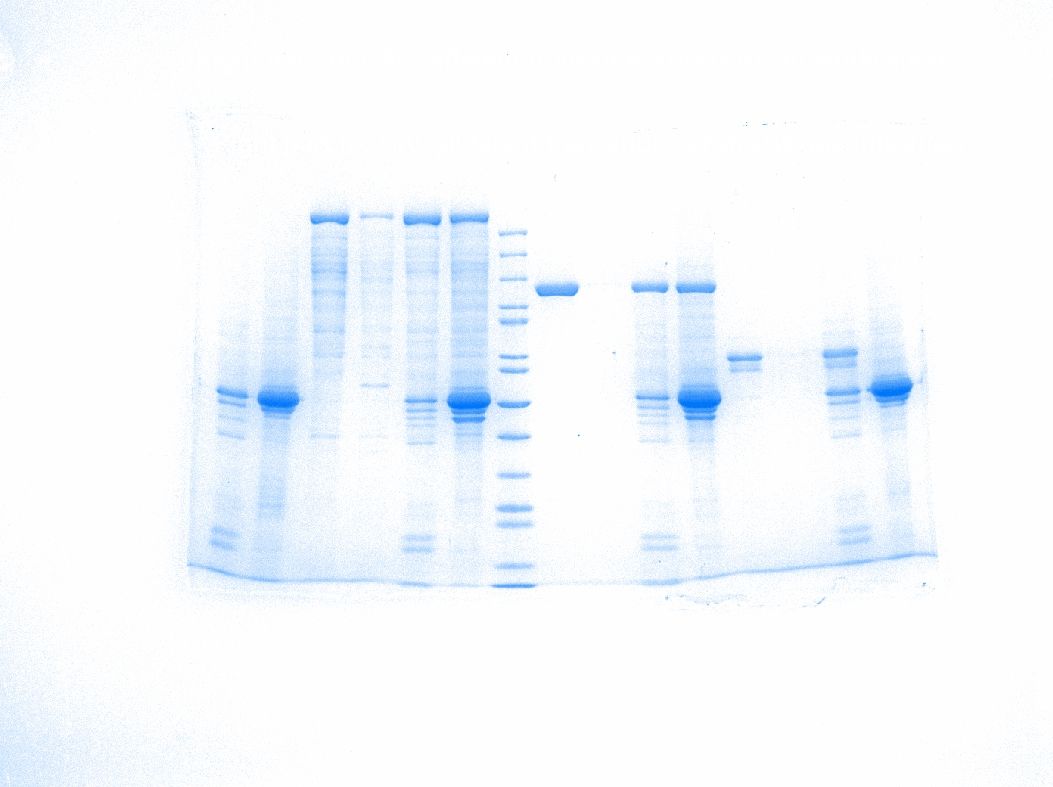

Supplement: Figure 7—source data 1. [file elife-86049-fig7-data1.zip › Figure 7-source data 1/Coomassie_Raw_GAC_Truncations_Factin_CoSedimentation_Right.jpg]
