# Supplementary material for: Structural and regulatory insights into the glideosome-associated connector from Toxoplasma gondii
Source: eLife. 2023 Apr 4;12:e86049. doi: 10.7554/eLife.86049 (PMC10125020; doi:10.7554/eLife.86049)
Supplement: Figure 5—source data 2. [file elife-86049-fig5-data2.pdf]

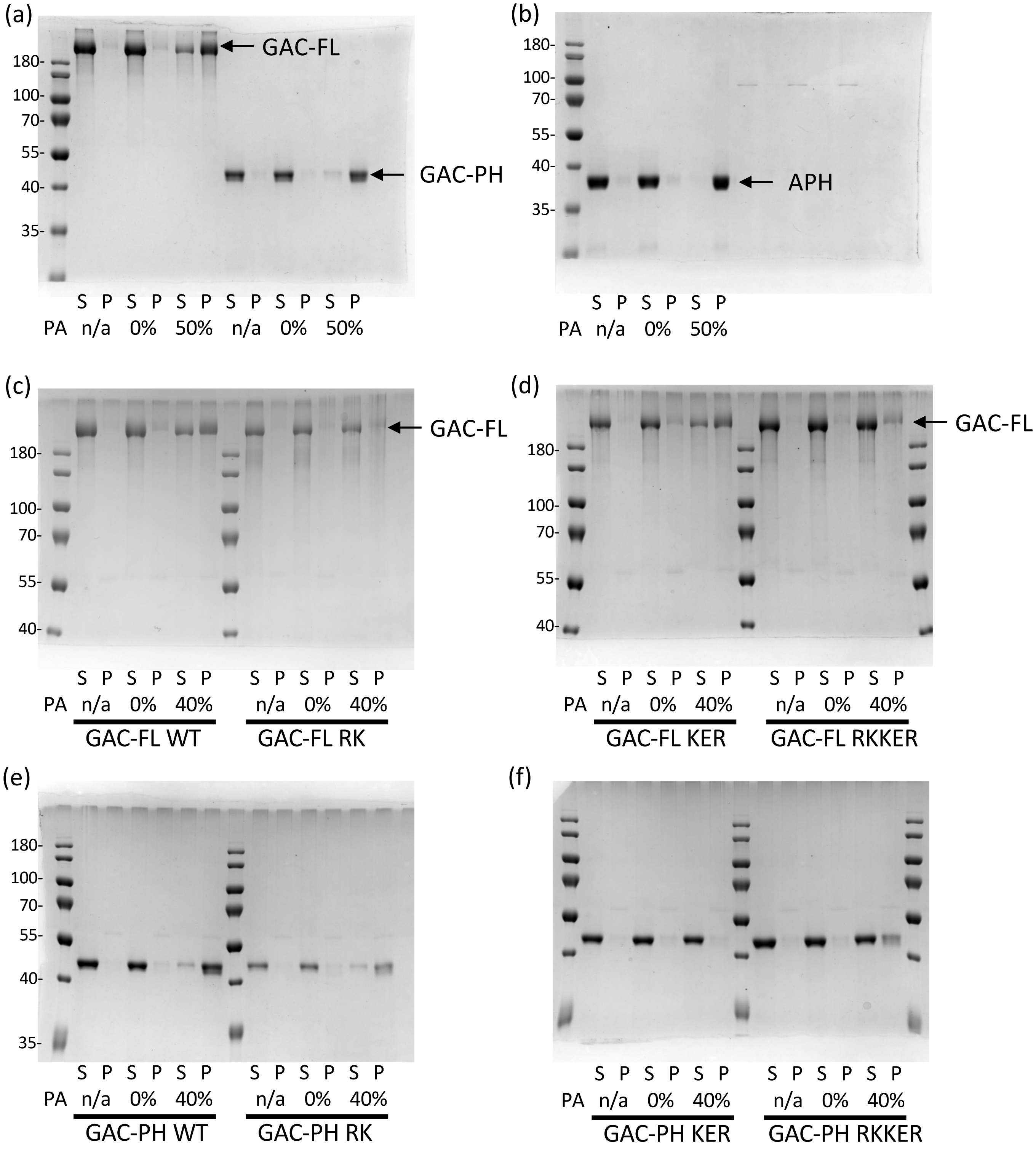

Figure 5 Source data 2: Liposome binding assays of PA binding by GAC and PH domain mutants *in vitro*. (a) SDS-PAGE gel for liposome binding assay with full-length TgGAC (GAC-FL) and the TgGAC PH domain (GAC-PH). (b) SDS-PAGE gel for liposome binding assay with TgAPH. (c & d) SDS-PAGE gel from liposome binding assays with full-length TgGAC-FL and the three mutated versions. (e & f) SDS-PAGE gel from liposome binding assays with full-length TgGAC-PH and the three mutated versions. n/a= no liposome. S=supernatant fraction after ultracentrifugation. P = pellet fraction after centrifugation. RK = RK/AA mutations. KER = KER/AAA mutations. RKKER = RK/AA + KER/AAA mutations. Key molecular weight markers indicated in kDa.
