# Supplementary material for: Structural and regulatory insights into the glideosome-associated connector from Toxoplasma gondii
Source: eLife. 2023 Apr 4;12:e86049. doi: 10.7554/eLife.86049 (PMC10125020; doi:10.7554/eLife.86049)
Supplement: Figure 7—source data 2. [file elife-86049-fig7-data2.pdf]

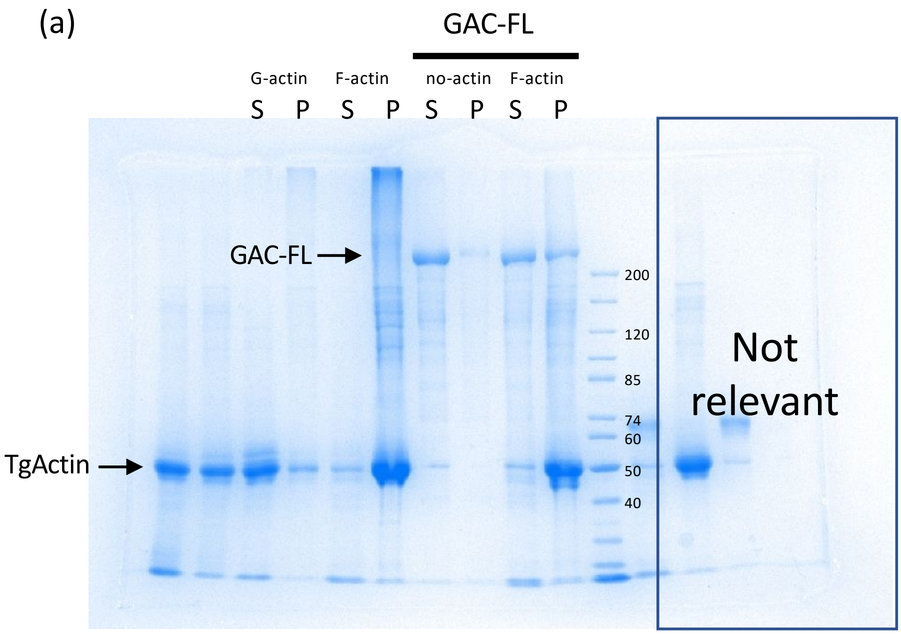

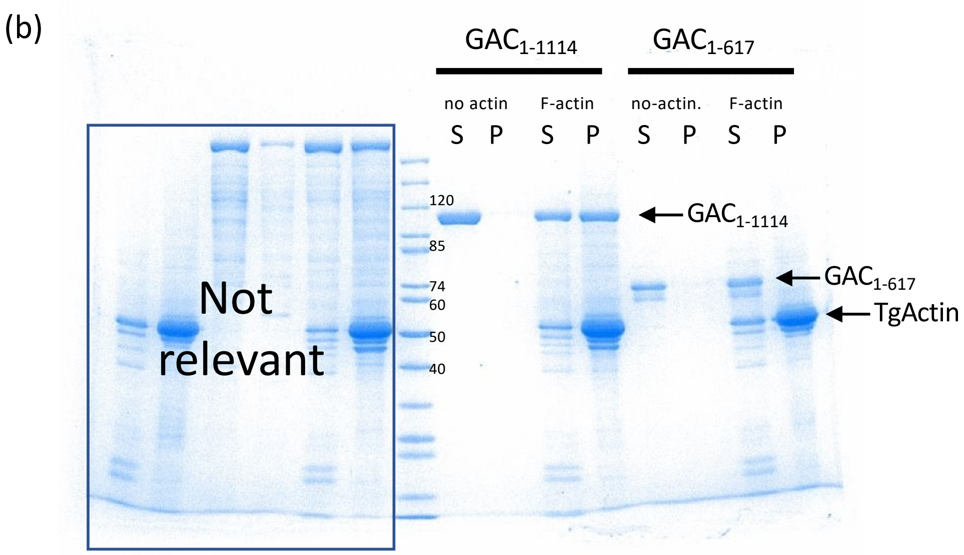

Figure 7 Source data 2: GAC interaction with TgActin filaments. (a) Coomassie-stained gel analysing full-length TgGAC (GAC-FL) remaining in supernatant or sedimenting upon centrifugation at 100’000 g. S: Supernatant. P: Pellet. (b) Coomassie-stained gel analysing truncated TgGAC ($TgGAC_{1\text{-}1114}$ and $TgGAC_{1\text{-}617}$) remaining in supernatant or sedimenting upon centrifugation at 100’000 g. S: Supernatant. P: Pellet. Key molecular weight markers indicated in kDa.
