# Supplementary material for: Structural and regulatory insights into the glideosome-associated connector from Toxoplasma gondii
Source: eLife. 2023 Apr 4;12:e86049. doi: 10.7554/eLife.86049 (PMC10125020; doi:10.7554/eLife.86049)
Supplement: Supplementary file 4. [file elife-86049-supp4.docx]

| **Sequence (5'-3')** | **Function** |
| --- | --- |
| AGCCGGCAGCAGTTGCTGCGAAGTTCACGATGTGCTTG | Forward for Q5mutagenesis to insert RK/AA point mutation in TgPHdomain (in pGST-TgPH plasmid) |
| CCTTCTTCGCTGCTGCTTTC | Reverse for Q5mutagenesis to insert RK/AA point mutation in TgPHdomain (in pGST-TgPH plasmid) |
| GTCAGGCGGCAGCCGACGCATTTGTCGAGATGATGGTGC | Forward for Q5mutagenesis to insert KER/AAA point mutation in TgPHdomain (in pGST-TgPH plasmid) |
| TCTTCGCGACCATGGGCAG | Reverse for Q5mutagenesis to insert KER/AAA point mutation in TgPHdomain (in pGST-TgPH plasmid) |
| CGCTGAAAGAGACTTGCGCCGCGACGCTCGAAGCCGCG | Forward for Gibson : TgPHdomain in pETHTB-GACwoPH-HIS6 plasmid (HindIII linearized) |
| CAGTGGTGGTGGTGGTGGTGCTCGATCACTTAAAAATTGTACGTTGCAGCGTCACGCC | Reverse for Gibson : TgPHdomain in pETHTB-GACwoPH-HIS6 plasmid (HindIII linearized) |
| GTGGATGGTACCAGATGGGC | Forward to put mutation in the endogenous locus (amplification of plasmid with mutations) |
| TCCGGTCGCGTCCTCAGGCGCAGCGGCCGGCACGAAGTGTGTTTCCTTTGTC | Reverse to put mutation in the endogenous locus (amplification of plasmid with mutations) |
| GTAAGAAACTGGGGATGTCCAGTTTTAGAGCTAGAAATAGC | Primer to generate gRNA (via Q5) targeting GAC Cter (intron) |
| TAAGGGCAGTCTCTGGAGTCGTTTTAGAGCTAGAAATAGC | Primer to generate gRNA (via Q5) targeting GAC 3'UTR |
| GATTCTTTGCCTGCCACCGAA | Reverse primer in GAC endogenous locus to screen clones for integration |
| CGTTTCTTCTTTTCTGATCTCCTC | Reverse primer in GAC endogenous locus to screen clones for integration |
| TGCCGCCATCTCTGGATTCC | Forward primer in GAC endogenous locus to screen clones for integration |

**Table:** Key DNA primers used generate mutations and the endogenous locus mutants for parasite experiments
